# Equity in the public social healthcare protection in Tanzania: does it matter on household healthcare financing?

**DOI:** 10.1186/s12939-023-01855-0

**Published:** 2023-03-20

**Authors:** Felician Andrew Kitole, Robert Michael Lihawa, Eliaza Mkuna

**Affiliations:** Department of Economics, Mzumbe University, P.O Box 5, Mzumbe, Tanzania

**Keywords:** Health financing, Health equity, Instrumental variable poisson, Health economics, Endogenous switching regression, Tanzania, Developing countries

## Abstract

Efforts to promote equity in healthcare involve implementing policies and programs that address the root causes of healthcare disparities and promote equal access to care. One such program is the public social healthcare protection schemes. However, like many other developing countries, Tanzania has low health insurance coverage, hindering its efforts to achieve universal health coverage. This study examines the role of equity in public social healthcare protection and its effects on household healthcare financing in Tanzania. The study used secondary data collected from the National Bureau of Statistics' National Panel Survey 2020/21 and stratified households based on their place of residence (rural vs. urban). Moreover, the logit regression model, ordered logit, and the endogenous switching regression model were used to provide counterfactual estimates without selection bias and endogeneity problems. The results showed greater variations in social health protection across rural and urban households, increasing disparities in health outcomes between these areas. Rural residents are the most vulnerable groups. Furthermore, education, income, and direct healthcare costs significantly influence equity in healthcare financing and the ability of households to benefit from public social healthcare protection schemes. To achieve equity in healthcare in rural and urban areas, developing countries need to increase investment in health sector by reducing the cost of healthcare, which will significantly reduce household healthcare financing. Furthermore, the study recommends that social health protection is an essential strategy for improving fair access to quality healthcare by removing differences across households and promoting equality in utilizing healthcare services.

## Introduction

Social health care protection is vital for the increase of health care utilization, decline in mortality and reduction of the household socioeconomic burden as a result of catastrophic health care spending [1–3]. Additionally, at times where countries are struggling to ensure health services are available to all people at lower costs and meet the sustainable development targets, the use of public health protection is unavoidable for major two purposes of reducing negative effects of excessive household health care financing and increase access to health care [4, 5]. Despite of potential initiatives put forward by governments in developed and developing countries towards relaxation of household pressure on the rising costs of health care through the establishment of more affordable public social health insurance schemes, there have been greater variations due to inequity in terms of subscriptions, accessibility and financial limitations across households and regions within countries [6–8].

Kitole et al. [5] acknowledges that “several studies have been made to analyze equity and equality towards access to healthcare globally and locally [9]. Despite these studies been a benchmark for the future studies they have had different and contradictory results which increases debates on whether issues of equity are important or can be achieved on health given that households and governments across the globe have different characteristics and level of development.” In this situation achieving equity in health care is a nightmare for most of the developing countries which are faced by deficit budget and heavily depends on donor and external assistance to finance health sector including Tanzania [5].

### Health Financing Reforms in Tanzania

Economic recession and budgetary burden made the government failing providing health services for free to all citizens. The failure was the doorway for the introduction of the user fee which in forms of cost sharing during 1990s. Table 1 presents series of health reforms made in a country since Arusha Declaration of Socialism and Self Reliance in swahili Ujamaa na Kujitegemea [1].

**Table 1:** Health sector reforms in Tanzania, 1967–2009

1967	Arusha Declaration	The Mwalimu Nyerere’s Arusha Declaration is the benchmark of major health reforms in Tanzania today which had motives of making health services to accessible to all people in the country particularly improving the livelihood of the marginalized groups
1977	Free Health Services	All private and profit oriented health services were banned and the government took the role of providing free health services to her people
1990	The introduction of First Tanzania’s Health Policy	
1991	Liberalizations of Private Health Care Facilities	
1991	Mixed Healthcare Financing	Tanzania government introduced a mixed system of health financing which involves the introduction of cost sharing policies
1993	Government/Development Partners Appraisal Mission on the Health Sector	
1993—1995	User Fees	i. Phase I: Introduction of user fees in refers and number of health services provided by regional hospitals ii. Phase II experienced the introduction of medical fees I regional hospitals in a country iii. Phase III introduction of the medical fees to all district hospitals iv. Phase IV experienced the introduction of user fees in health centres and dispensaries
1995	The first Plan for Health Sector Reforms Agreement to engage in SWAP programs in Health	
1996	Community Health Fund (CHF)	This is a form of insurance plan under the government authorities which was designated purposely or the rural populations in a country. It was established in order to increase marginalized groups and large population employed in informal sectors to have equal access to health services and lower number of death due to disease
1998	Agreement to enter a SWAP program in Health	
1999	Health being introduced in Poverty Reduction Strategy (PRS) as a matter of priority	
1999	Introduction of Health Busket Fund	
1999	Introduction of National Health Insurance Fund (NHIF) but its operation began in 2001	
2000	National Package of Essential Health Interventions Approved	
2002	Introduction of Private insurance schemes	
2003	Health Sector Strategic Plan II (HSSP II)	
2004	Introduction of emergency infrastructure rehabilitations programs	
2005	Introduction of Tanzania Essential Health Intervention Project (TEHIP)	
2006	Establishment of the Tanzania Joint Assistance Strategy	
2007	Social Health Insurance Benefit (SHIB)	
2007	The National Health Policy 2007	
2008	Establishment of the Health Sector Human Resource Strategic Plan	
2009	The announcement for Health Sector Strategic Plan IIII (HSSP III)	
2015	Health Sector Strategic Plan IV (HSSP IV)	

Source: Ministry of Health and Social Welfare (MHSW) [10] and Andrew [1]

### Health Insurance in Tanzania

Literature shows that Tanzania is still lagging behind in the universal health care coverage which is highly perpetuated by the low proportion of the Tanzania who have been subscribed to health insurances. In 2019, only 32% had health insurance covered whereas majority 26% are subscribed to Community Health Fund (CHF), 8% subscribed to National Health Insurance Fund (NHIF) and only 1% of all Tanzania were subscribed to other private health insurance schemes [1, 11]. CHF mainly focusses its coverage in the rural population while private health insurances targets most of the urban population [11].

In Tanzania, the health insurance policy has been implemented for a long time than any of the current operated health financing scheme; Act of Parliament No. 8 of 1999 established the today used NHIF and started is operations in June 2001 [1, 10, 11]. Through the NHIF, public formal sector employees are paying a are required to contribute 3 percent of a monthly’s salaries while government also contribute same share to make a total of 6% with not more than 5 other members of the family as a dependent [8, 12, 13].

National Health Insurance Fund (NHIF) is the authorized government health insurance in Tanzania. Membership to this insurance agency is unavoidable for all workers in the formal employment while for different informal sectors’ workers it is voluntarily. NHIF covers the healthcare costs for inpatient based on the approved rates, while any amount the patient or insured person top up above the NHIF coverage is known as the copayment or coinsurance. Therefore, the financial security provided by NHIF isn’t comprehensive enough and inadequate for all patients who seek healthcare services in private, or in both public and private facilities as result of exclusion in health insurance [14].

On the other hand, despite the introduction of CHF in 1996 which mainly targeted rural and other informal sectors, the scheme has been underperforming and this is justified by considering the coverage of the scheme which have remained to be very low for a long time while the enrolment being far below the target of HSSP III. Studies in Tanzania have shown that some members have been pulling out from the schemes which have led to a slow enrolment trend and lowering government efforts towards universal health coverage [11].

As a remedy to CHF the government decided to introduce Social Health Insurance Benefit (SHIB) in 2007. SHIB is component of National Social Security Benefits (NSSB) that was established lately 2007 of which all members of the NSSF have access to medical care through SHIB after undergoing registration process with only one facility of their choice. This scheme accredits both private and public health care providers in the country [4, 15].

Most of the urban population in Tanzania are covered with health insurance compared to those in rural areas despite most of the health insurances schemes in these areas being available at low costs compared to those in urban counterparts [5, 9]. This makes more private and newly established health insurance schemes to pile up in urban areas due to the good and competitive market. As a result, even the public social health protection schemes are highly concentrated in urban and rarely found in rural areas hence creating huge gap between rural and urban households towards social health inequity. Studies [4, 7, 9] suggest that households’ socioeconomic characteristics and institutional arrangements are major factors towards breaking the social health protection inequity challenges.

The affordability, quality, effectiveness and efficiency of healthcare largely depends on the ability of healthcare financing mechanism within health sector. Thus, low insurance coverage leads to over-reliance on the direct payments at the point of use of health care, which is among the fundamental problem that restrains most developing countries towards realization of the universal health coverage. This is perpetuated with the fact that, direct payments lead to higher levels of inequity and normally denying poorest households (population) access to the health care when needed [16, 17].

Dake [16] argued that social health protection (SHP) has been identified as a strategy for achieving universal access to healthcare by the International Labor Organization who define social health protection as a series of public or publicly organized and mandated private measures against social distress and economic loss caused by the reduction of productivity, stoppage or reduction of earnings or the cost of necessary treatment that can result from ill health. Therefore, these are deliberate interventions to increase health care accessibility in Tanzania and may other developing countries be largely destructed by mechanisms to which government and household finance health sector.

Tanzania has a long way to go not only in realizing UHC as a global agenda but achieving country’s dream of ensuring health care becomes a primary right for every citizen by making the service accessible to all people at lower costs and minimal inconveniences [1]. In this regard, there is a need for an equity analysis to identify major socioeconomic and institutional characteristics that may lead to the expulsion of certain groups of people from accessing health care services which this will be a stepping stone towards realization of the long-awaited dream of equity in health. Uniqueness of the current study is brought by the fact that it examines simultaneously effects equity on the health insurance coverage and its effects on household health care financing, unlike many studies which have examine these two separately [5, 9, 14] and ignoring the fact government efforts in moving towards universal health coverage can widen health inequity across households if the two will not be properly addressed.

## Methods and data

This paper utilizes secondary data from the 2020/21 Tanzania National Panel Survey (NPS). The National Panel Survey (NPS) is a nationally representative longitudinal survey designed to provide data from the same households over time in an attempt to better track national and international development indicators, understand poverty dynamics and welfare, and to evaluate policy impacts in the country. It contains information on the general health and household health insurance statuses. The 2021 round of the GDHS included a national module in which myriad national policies including the NHIS were assessed. This paper combines data from questions on coverage under NHIS with demographic and socio-economic indicators to analyze equity on the health insurance coverage in Tanzania and its effects on the household healthcare financing. The choice of the dataset is based on the fact that Tanzania is currently heading towards the end of its 25 years development vision (2000 – 2025) therefore these data (NPS 2021) will have the most accurate information heading 2025 making our estimates more realistic for the twenty-five years reflections [5, 18].

### Definition and measurement of variables

In examining equity on social health protection, the dependent variables used in this study was the status of household coverage with health insurance of which the public health insurance schemes (NHIF and CHF) were used as the indicator of the insurance coverage due to the fact that they are easily available and cheap comparing to private schemes. Therefore, this is binary information in nature whose measurement and treatment requires the use of the limited dependent variable choice models [9, 19]. On the other hand, on examining the determinants of health equity among households the multiple response categories have been used to classify households under different four levels of health equity. The last objective is to examine on how social health protection schemes influences equity on households’ health care financing in Tanzania. In this part, the dependent variable used is the household health care expenditure which is the amount of money that household spends on health. Other variables used in this study have been described at Table 2.

**Table 2:** Measurement and description of variables

**S/N**	**Variable name**	**Measurement**	**Expected sign**		
			**Insurance**	**Equity**	**Healthcare financing**
01	Sicknesses	Households reported having any sickness month before surveys (Dummy, 1 = Sickness, 0 otherwise	Negative	Positive	Positive
02	Residence	Dummy, 1 = Urban, 0 otherwise	Positive	Indeterminate	Indeterminate
03	Household size	Total number of members in a household	Positive	Intermediate	Indeterminate
04	Education level	Dummy, 1 = primary, 0 otherwise	Indeterminate	Negative	Negative
05	Sex	Dummy, 1 = male, 0 otherwise	Uncertainty	Uncertain	Uncertain
06	Distance to health facility	Distance in kilometers	Uncertain	Uncertain	Uncertain
07	Education	Total number of years of schooling	Indeterminate	Negative	Negative
08	Age	Age in years	Uncertain	Uncertain	Uncertain
09	Presence of NCD	Dummy, 1 = For presence of any NCD, 0 otherwise	Negative	Positive	Positive
10	Out of pocket payment	Total cost incurred by household seeking health services	Uncertainty	Positive	Positive
11	Employment	Dummy, 1 = employed, 0 otherwise	Positive	Negative	Negative
12	Marital status	Dummy, 1 = married, 0 otherwise	Negative	Uncertain	Uncertain
13	Dependence ratio	Ratio of the non-working household members to total members of household	Negative	Negative	Negative
14	Income	Total household monthly income	Positive	Positive	Positive
15	Asset	Dummy, 1 = Asset ownership status (motorcar, bicycle, land etc.), 0 otherwise	Positive	Negative	Negative

### Analytical modelling

#### Determinants of health insurance subscription, stratified by residence

In this section, the study employed logit regression model of which the outcome variable ${y}_{i}$ is binary assuming only two values that for convenience we have coded them as zero or one. Thus, the expression is defined as;

$$y_i=\left\{\begin{array}{l}1\;if\;the\;i-th\;household\;have\;health\;isurance\\0\;otherwise\end{array}\right.$$

We view ${y}_{i}$ as a realization of a random variable ${Y}_{i}$ that can take the values one and zero with probabilities ${\pi }_{i}$ and $1 - {\pi }_{i}$ respectively. The distribution of ${Y}_{i}$ is called a Bernoulli distribution with parameter ${\pi }_{i}$ and can be written in compact form as;

$$Pr\left\{{Y}_{i}={y}_{i}\right\}={\pi }_{i}^{{y}_{i}}{(1 - {\pi }_{i})}^{1-{y}_{i}}$$

Whereas, ${y}_{i} = 0, 1$ given that if ${y}_{i}=1$ we obtain ${\pi }_{i}$ and when ${y}_{i}=0$ we obtain $1 - {\pi }_{i}$. No, let ${\pi }_{i}$ be a linear function of the covariates with $\beta$ as a vector of regression coefficient of which equation …. (below) is referred as the linear probability model.

$${\pi }_{i}={x}_{i}^{^{\prime}}\beta ,$$

Through the imposition of the complex restriction on the coefficients, the simple solution to this challenge is obtained through transformation of the probability to remove the range restrictions [20], and the model the transformation as a linear function the covariate, which can be done in two steps, starting with moving probability ${\pi }_{i}$ to the odds which is defined as the ratio of the probability to its complement, or the ratio of favorable to unfavorable cases;

$${odds}_{i}=\frac{{\pi }_{i}}{1-{\pi }_{i}}$$

At the second step, the logarithms are used to estimate logit or the log of odds which has the effect of removing the floor restriction. To see this point note that as the probability goes down to zero the odds approach zero and the logit approaches $-\infty$

$${\eta }_{i}=logit({\pi }_{i})=log\frac{{\pi }_{i}}{1-{\pi }_{i}}$$

At the other extreme, as the probability approaches one the odds approach $+\infty$ and so does the logit. Thus, logits map probabilities from the range (0, 1) to the entire real line. Therefore, solving for the ${\pi }_{i}$ from equation …. gives;

$${\pi }_{i}={logit}^{-1}({\eta }_{i})=\frac{{\mathrm{e}}^{{\eta }_{i}}}{1+{\mathrm{e}}^{{\eta }_{i}}}$$

### Determinants of health equity among households in Tanzania

The ordered logistic model was used to examine determinants of equity across households in Tanzania based on their localities or residences as described in the study that household premises (rural or urban) can reduce government efforts of ensuring equitable health and universal coverage in a country due to socioeconomic and institutional differences and advantage existing between these two areas.

The model follows that;

$$\begin{array}{ccc}y^\ast=\beta'x_i+\varepsilon_i&&-\infty<y_i^\ast<\infty\end{array}$$

Whereas ${\mathrm{y}}_{\mathrm{i}}^{*}$ represents levels of Equity, ${\beta }^{^{\prime}}$ is a vector of parameters that should be estimated, ${x}_{i}$ is an observed vector of non-random explanatory variable, which shows the characteristic of ${\mathrm{i}}^{\mathrm{th}}$ Variable and ${\varepsilon }_{i}$ presents error term which is logistically distributed. Since ${\mathrm{y}}_{\mathrm{i}}^{*}$ is a latent variable, standard regression techniques are not applicable to estimate the sample size. If ${\mathrm{y}}_{\mathrm{i}}$ is considered as a discrete and observable variable which shows different levels household equity, the relation between latent variable ${\mathrm{y}}^{*}$ and observable variable ${y}_{i}$ is obtained from ordered logit model as follows:

$$\begin{array}{cccc}y_i=1&if&-\infty\leq y_i^\ast<\mu_1&i=1,\dots,n\\y_i=2&if&\mu_1\leq y_i^\ast<\mu_2&i=1,\dots,n\\y_i=3&if&\mu_2\leq y_i^\ast<\mu_3&i=1,\dots,n\\y_i=J&if&\mu_{j-1}\leq y_i^\ast<+\infty&i=1,\dots,n\end{array}$$

In which ʹ*n*ʹ is the value for the sample size, ʹµʹ and ʹ*s*ʹ are the thresholds that define observed discrete answers and should be estimated. The probability of ${\mathrm{y}}_{\mathrm{i}}=\mathrm{j}$ should be calculated by the following relation:  

$$\mathrm{Pr}\left({\mathrm{y}}_{1}=\mathrm{J}\right)=\mathrm{Pr}({\mathrm{y}}_{1}\ge {\upmu }_{\mathrm{j}-1})=\mathrm{Pr}({\upvarepsilon }_{1}\ge {\upmu }_{\mathrm{n}-1}-\upbeta {\mathrm{x}}_{1})=\mathrm{F}(\upbeta {\mathrm{x}}_{1}-{\upmu }_{\mathrm{j}-1})$$

In cumulative probability expression, ordered logit model estimates the likelihood of person ʹ*I*ʹ to be at ʹj^th^ʹ level or less $(1\dots ,\mathrm{ j}-1)$. It should be noted that the answer groups in ordered logit model are ordered. Ordered logit model is expressed as follows:

$$log\left[\frac{{y}_{j}({x}_{i})}{1-{\gamma }_{i}({x}_{i})}\right]={\mu }_{j}-\left[{\beta }_{1}{x}_{1i}+{\beta }_{2}{x}_{2i}+\dots +{\beta }_{k}{x}_{ki}\right]$$

Whereby $$\mathrm{j}=1\dots ,\mathrm{ J};\mathrm{ I}\dots ,\mathrm{ n}$$

In which, ${\mathrm{y}}_{\mathrm{j}}$ is a cumulative probability of the following:

$${y}_{j}\left({x}_{i}\right)=y\left({\mu }_{j}-{\beta }^{^{\prime}}{x}_{i}\right)=p\left({y}_{i}\le j|{x}_{i}\right)$$

${\upbeta }^{\mathrm{^{\prime}}}$ is the column vector and of ${\upbeta }_{1}, {\upbeta }_{2}\dots . {\upbeta }_{3}$ parameters and ${\mathrm{x}}_{\mathrm{i}}$ is the column vector of explanatory variables. ${\upmu }_{\mathrm{j}}$ is only dependent on probability of predicting category and is not dependent on explanatory variables unlike the independent part described in the following expression that:

$${\beta }_{1}{x}_{1i}+{\beta }_{2}{x}_{2i}+\dots +{\beta }_{k}{x}_{ki}$$

These two characteristics guarantee that the answers groups are ordered and show that the results are a series of parallel lines. Parameters are estimated by maximum likelihood estimation method, which maximizes the probability of categorization. The calculation of the marginal effect of one unit in ${\mathrm{x}}_{\mathrm{k}}$ predictor on the probability of ʹ*j*ʹ category is as follows:  

$$\frac{\mathrm{\delta P}({\mathrm{y}}_{\mathrm{i}}=\mathrm{j}|{\mathrm{x}}_{\mathrm{i}})}{\updelta {\mathrm{x}}_{\mathrm{k}}}=\left[\frac{\mathrm{\delta y}({\upmu }_{\mathrm{j}}-{\upbeta }^{\mathrm{^{\prime}}}{\mathrm{x}}_{\mathrm{i}}}{\updelta {\mathrm{x}}_{\mathrm{k}}}-\frac{\mathrm{\delta y}({\upmu }_{\mathrm{j}-1}-{\upbeta }^{\mathrm{^{\prime}}}{\mathrm{x}}_{\mathrm{i}})}{\updelta {\mathrm{x}}_{\mathrm{k}}}\right]=\left[\upsigma ({\upmu }_{\mathrm{j}-1}-{\upbeta }^{\mathrm{^{\prime}}}{\mathrm{x}}_{\mathrm{i}})-\upsigma ({\upmu }_{\mathrm{j}}-{\upbeta }^{\mathrm{^{\prime}}}{\mathrm{x}}_{\mathrm{i}}){\upbeta }_{\mathrm{k}}\right]$$

whereas

$${\mu }_{j}=+\infty , {\mu }_{*}=-\infty ,{\sigma }_{j}\left({x}_{i}\right)=\frac{\delta {y}_{i}({x}_{i})}{\delta {x}_{k}}$$

Making decisions about using variables’ value in estimation is very important, because the marginal effect depends on the values of all independent variables. Since total probability always equals to 1, then the total marginal effect for each variable equal to 0. Not only that, but also it should be noted that the marginal effect is not direct binary variable and it can be obtained by calculating the difference between the two possible probabilities. Therefore, in this study the ordered equity levels under examination have been described as;

$$y^\ast=\left\{\begin{array}{lll}High\;Equity&if&y_i=1\\Moderate\;Equity&if&y_i=2\\Low\;Equity&if&y_i=3\end{array}\right.$$

### Effects of social health protection equity on household health care financing

In this section the study employed the endogenous switching regression (ESR) to model effects of social health protection towards equitable household health care financing in Tanzania. Most studies in health have been widely exposed to the instrumental variable models due to the possible endogeneity arises during estimating health effects.

It is assumed that the households consider the benefit equitable social health insurance through the health care expenditure derived from household health care financing pattern. The following model specifies the selection equation ${P}^{*}$ where ${P}^{*}$ is the latent variable which is not observed. ${P}^{*}$ can, however, be expressed as a function of some observed health, household and institutional characteristics.

$$\begin{array}{c}P^\ast=\alpha Z_i+\mu_i\\I_i=1\;\mathrm{if}\;P^\ast>0\;\mathrm{and}\;I_i=0\;\mathrm{if}\;P^\ast\leq0\end{array}$$

${I}_{i}$ is a binary variable which takes a value of 1 for household with health insurance coverage and 0 for those who do not have health insurances. ${Z}_{i}$ represents factors that affect the household decision to subscribe to social health protection (insurances), $\alpha$ denotes the vector of parameters indicating the magnitude and direction of each explanatory variable’ s effect on the decision on household to subscribe to social health protection. The residual ${\mu }_{i}$ captures the unobserved factors and measurement errors.

The two regimes that the households fall into are represented by the following two regression equations.

$$\begin{array}{cccc}Regime\;1:& {Y}_{1i}={\beta }_{1}{X}_{i}+{\varepsilon }_{1i} & \mathrm{if}& {I}_{i} = 1\\ Regime\;2:& {Y}_{2i}={\beta }_{2}{X}_{i}+{\varepsilon }_{2i}& \mathrm{if}& {I}_{i} = 0\end{array}$$

${Y}_{1i}$ and ${Y}_{2i}$ are the dependent outcome variables (i.e., household health expenditure) determined by the exogenous variables ${X}_{i}$, ${\beta }_{1}$, and ${\beta }_{2}$, are parameters that show the direction and strength of the relation between the outcome variable and the independent variables. ${\varepsilon }_{1i}$ and ${\varepsilon }_{2i}$ are error terms.

## Results

Results in Table 3 which explains different household socioeconomic characteristics related to the health care financing show that the average monthly household out of pocket expenditure in Tanzania for the period of 2020/21 was Tanzania shillings (Tshs) 996,893 of which the household with the least out of pocket expenditure in the given time period incurred a total expense of Tshs. 38,800 which is just 86.22 percent of entire least income earned by a household meaning that poor households are highly hurt with the OOP compared to higher income earners. On the other hand, households use an average of 5.38 km to seek for medical health care of which the nearest household to health facility use just 0.2 km while those in far areas spend more than 28 km.

**Table 3:** Descriptive statistics of variables included in the income equation

**Variable name**	**Mean**	**Std Deviation**	**Minimum**	**Maximum**	**T test**
Household size	6.3854	2.8616	1	38	2.674**
Years of experience	9.4838	8.0037	1	35	3.451**
Distance to facility	5.3802	3.65882	0.2	28.5	1.052
Out of pocket expenditure	996,893	2343936	38800	10,500,000	8.250***
Years of schooling	7.8946	4.3189	0	21	4.113**
Age	47.594	15.7342	19	102	-0.351
User fee	160,660	148,132	14,600	4,614,256	9.305***
Total Health expenditure	455,000.1	1,211,302	4,325	31,500,320	1.021
Dependency ratio	6.4051	4.6721	0.652	28.672	-4.251***
Household income	936,540	2331422	45000	154,005,420	4.563**

^***^ p < 0.01, ** p < 0.05, * p < *0.1*

Moreover, results in Table 3 show that an average household size was 6.38 while the household with highest number of the family size had 38 members. Notwithstanding that, an average household dependency ratio was 6.4 implying that the household working members have higher burden to support the non-working members of the household. However, a household with the least dependency ratio had just 0.652 and the one with the highest burden had 28.672. On top of that the average age across all households in Tanzania was 47.59 years with oldest having 102 years.

**Fig. 1:**
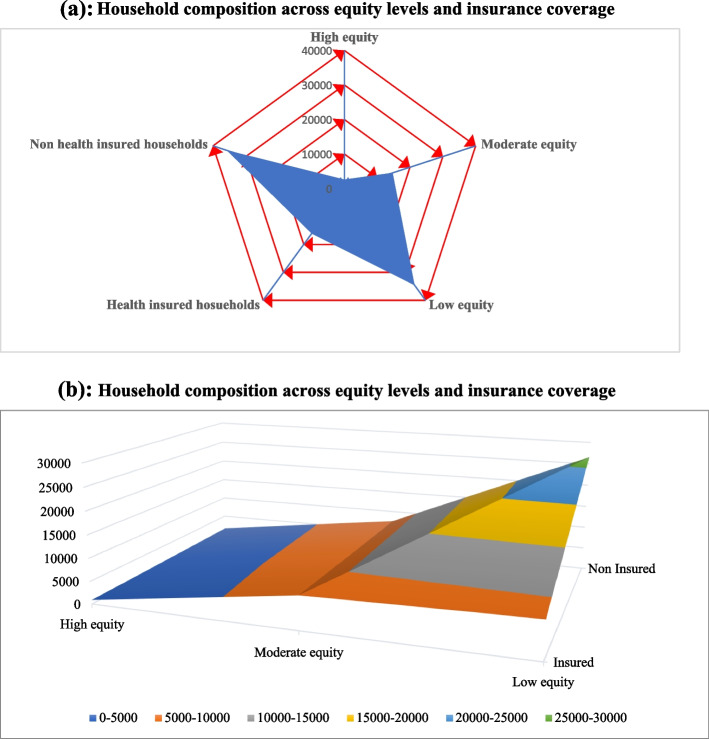
**a** Household composition across equity levels and insurance coverage. **b** Household composition across equity levels and insurance coverage

Results on Fig. 1(a) and (b) shows that most households are found under low equity level of the health as the curve inclined more towards low equity compared to moderate and high equity levels. On the other hand, the blue curve has highly inclined towards non health insured household implying that most households in Tanzania do not have health insurance hence not protected under the social health protection making them vulnerable to impoverishment as the result of health expenditures. Comparatively, results on Fig. 1(b) show that most of the non-insured households are found under low level of equity.

**Fig. 2:**
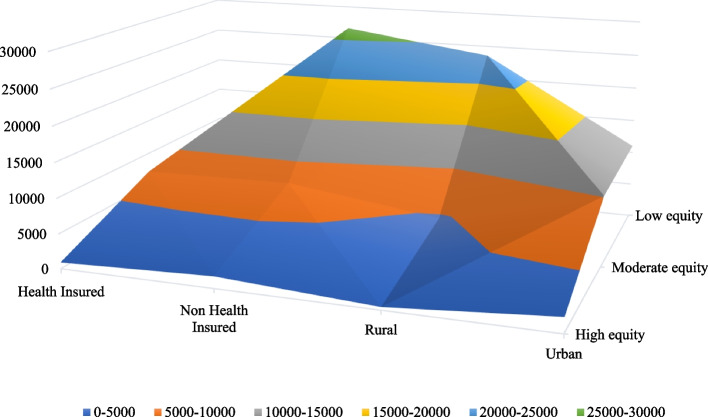
Household social health protection composition based on residence

Moreover, results on Fig. 2 implies that there are great variations of equity on social health protection across households in rural and urban areas in Tanzania. Most households residing in rural areas little enjoying economies of social health protection compared to their urban counterparts. This is justified by fact that only 16.27% out of 2,587 households under the high were households living in rural areas while the majority were living in urban areas. Thus, the concentration of households in low equity were mostly found to live in rural areas (68.16%) and only few (31.84%) were living in urban areas.

### Determinants of health insurance subscription, stratified by residence

Results in Table 4 presents estimates on the determinants of health insurance subscription among households in Tanzania who were stratified based on the residences (rural vs. Urban). The aim of making this stratification is based on the fact that households in rural and urban areas are heterogeneous in most of the socioeconomic characteristics and their level of economic interaction is quite antagonistic with most of the urban residents being more well-off compared to rural residents.

**Table 4:** Determinants of social health protection (Insurance coverage)

**Variable**	**Rural**	**Urban**	**Pooled**
	**Marginal effects (dy/dx)**		
Sex	0.437 (0.583)	-0.124*** (0.003)	-0.096** (0.008)
Age	0.007*** (0.000)	0.214*** (0.021)	0.129*** (0.016)
Household size	-0.342* (0.109)	0.168*** (0.005)	0.188*** (0.016)
Marital status	0.274** (0.013)	-0.109** (0.018)	0.181* (0.075)
Education	0.041** (0.002)	0.162*** (0.002)	0.098*** (0.000)
User fee	-0.205** (0.000)	-0.128*** (0.000)	-0.103*** (0.005)
Out of pocket	-0.341* (0.137)	-0.118*** (0.015)	-0.109*** (0.003)
Household income	0.099** (0.045)	0.266*** (0.102)	0.392** (0.092)
Distance to health facility	-0.242* (0.094)	-0.357*** (0.090)	-0.1491*** (0.002)
Employment	0.362*** (0.009)	0.105*** (0.021)	0.195* (0.003)
Sickness	0.083* (0.032)	0.207*** (0.012)	0.095*** (0.017)
Household dependence ratio	-0.340* (0.112)	-0.144*** (0.002)	-0.191** (0.049)
Number of observations	23,562	11,005	34,567
Prob > chi squared	0.0000	0.0006	0.0072
Pseudo R squared	0.3782	0.3954	0.4024

*Standard errors in parentheses*

^***^ p < 0.01, ** p < 0.05, * p < *0.1*

Results in Table 4 show that the likelihood for a household to be covered by health insurance declines as such household incurs extra health care costs though out of pocket payments by 34.1 percent in rural areas, 11.8 percent in urban areas and 10.9 percent at the national level. Moreover, user fees which are normally charged by health facilities during medical consultations have been found to lower the likelihood of household to be covered by health insurance significantly. These results are different from other studies [15, 21, 22] which found user fees to be a burden hence accelerate household to opt for the health insurances.

In addition to that, results in Table 4 show that sex of the head of household is irresponsiveness towards household health insurance coverage status in rural areas but responsive to urban areas. Specifically, results show that being male reduces the likelihood of urban household being covered with health insurance significantly by 12.4 percent compared to female headed households in urban areas. Moreover, at the national level it reduces the likelihood of household being covered by 9.6 percent significantly. These results are similar to most studies [23–25] in developing countries which showed that sex of the head of household has no effects on the household health insurance coverage status.

Other demographic factors that were found to be significant across all clusters (i.e., rural, urban and country-wise) include age, household size and marital status. These results are supported by the theory of the demand for health and the health belief model which argues that household demographic characteristics are key components explaining household members decision toward demand for health and health insurance [26]. Therefore, having theoretical support this implies that these results can be used for the practical test in developing countries in order to facilitate government efforts towards increasing health care coverage and meet in-country and international health targets [24, 27, 28]. Additionally, results reveal that an increase in years of schooling or having higher education increase the likelihood of household to be covered by health insurance significantly at 4.1 percent in rural areas, 16.2 percent in urban areas and 9.8 percent at country level. Studies [19, 29] enlighten that classroom education alone cannot influence households’ decision to pay for health insurances rather having relevant knowledge and information on the importance of health insurances.

### Determinants of health equity among household in Tanzania

**Fig. 3:**
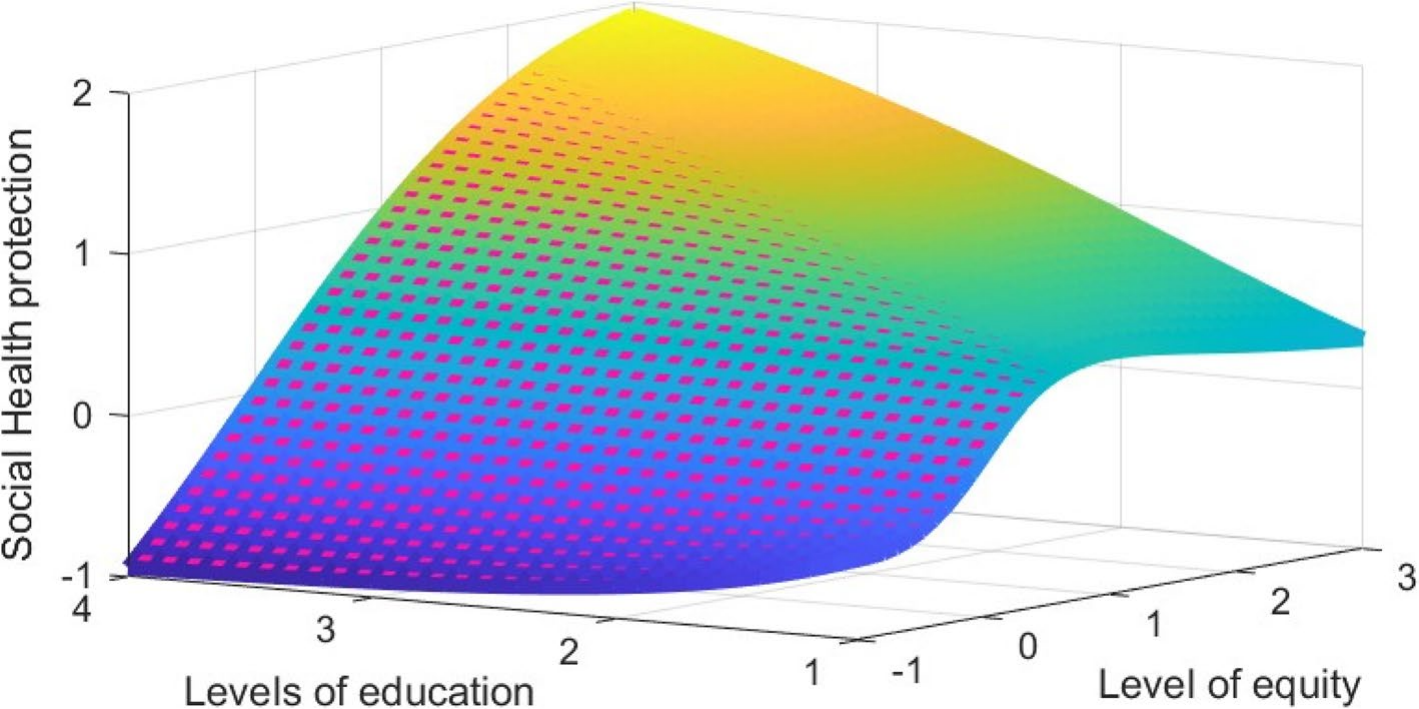
Social health protection, equity and education across households

Results on Fig. 3 justifies the importance of education in enhancing the widespread of the social health protection subscription (adoption) among households in Tanzania. Similar results were found by Binyaruka and Borghi [6] and Kitole et al. [5] who argued that most people in urban areas are more likely to subscribe to social health protection programs not because of any other economic advantages, rather they are more education and information advantage compared to the rural residents. Having right and timely information is key towards behavioral change which influences more individual decision to invest in his or her health [14, 16]. Moreover, as household social protection statuses increases the household equity level were also found to increases implying that social protection are necessary to achieve equity in health.

On the other hand, results in Table 5 presents the ordered logit regression estimates that explains the determinants of household health equity across three different perceived levels of equity (i.e., low equity, moderate equity and high equity) for both rural and urban areas in Tanzania for the period of 2020/21. Thus, results show that household demographic characteristics such as sex (dummy male), age of the head of household, number of the family members in a household and marital status have significant relationship to the health equity among household in Tanzania, implying that any plan to improve health equity should take into consideration characteristics and compositions of households in order to increase its likelihood of successfulness [26, 30].

**Table 5:** Ordered logit regression on determinants of health equity among households in Tanzania

**Variable**	**Coefficients**	**Three levels of equity (Rural)**			**Three levels of equity (Urban)**		
		**Low equity**	**Moderate equity**	**High equity**	**Low equity**	**Moderate equity**	**High equity**
Sex	-0.332*** (0.020)	-0.112** (0.016)	-0.140** (0.018)	-0.195* (0.080)	-0.174* (0.008)	0.155** (0.014)	0.140** (0.011)
Age	0.300 (0.414)	0.321 (0.582)	0.047 (0.109)	0.285 (0.261)	0.056* (0.016)	0.097** (0.022)	0.069** (0.018)
Household size	-0.002*** (0.000)	0.248 (0.103)	0.082** (0.005)	0.009** (0.001)	0.145** (0.040)	0.084** (0.004)	-0.109** (0.010)
Marital status	-0.011 (0. 802)	-0.217** (0.028)	0.084** (0.004)	0.168** (0.032)	-0.225* (0.100)	0.125** (0.020)	0.196** (0.007)
Education	0.198** (0. 022)	-0.371** (0.081)	0.097* (0.034)	0.153*** (0.000)	-0.278* (0.101)	0.109** (0.016)	0.237** (0.098)
User fee	-0.066*** (0.008)	0.110** (0.028)	-0.040** (0.002)	-0.259** (0.101)	0.209** (0.089)	-0.107* (0.013)	-0.250** (0.072)
Out of pocket	-0.249*** (0.009)	0.289** (0.004)	-0.015** (0.003)	-0.089** (0.007)	0.361** (0.100)	-0.052* (0.010)	-0.217** (0.016)
Household income	0.180*** (0.004)	-0.381** (0.125)	0.140*** (0.011)	0.209** (0.026)	-0.179** (0.023)	0.114*** (0.001)	0.230*** (0.072)
Distance to health facility	-0.063** (0.010)	0.131** (0.009)	0.114 (0.137)	0.044* (0.011)	0.108*** (0.000)	-0.193** (0.014)	-0.215** (0.022)
Employment	0.181*** (0.011)	-0.408** (0.117)	0.099* (0.024)	0.126** (0.007)	0.104* (0.010)	-0.180** (0.035)	0.122** (0.003)
Sickness	-0.210*** (0 .008)	0.165*** (0.002)	-0.015 (0.188)	-0.151 (0.163)	0.005** (0.001)	-0.126** (0.026)	-0.195** (0.019)
Household dependence ratio	0.107*** (0.016)	0.214** (0.069)	-0.002 (0.186)	-0.294** (0.114)	0.049** (0.025)	-0.063** (0.013)	-0.091** (0.0029)
Number of observations					34,567		
Prob > chi squared					0.0000		
Pseudo R squared					0.3602		

*Standard errors in parentheses*

^***^ p < 0.01, ** p < 0.05, * p < *0.1*

Nonetheless, results in Table 5 show that out of pocket payments and user fees increase the likelihood of most households in Tanzania to fall under low equity level significantly for households in both urban and rural areas. These findings are similar to several studies [31, 32] in developing countries which argues that an increase in the health care out of pocket payment in most of the developing countries’ residents distort their abilities to seek for the modern health acre and hence start diverging to the traditional health service which lowers health care equity across countries and between households [33]. Another notable factor that deteriorates equity among households in Tanzania includes diseases (sickness) and the distance of the health facilities from households’ residents which describes time taken by sick household members to get medical care. Similar factors were described in studies [5, 15, 16] which describes that when health facilities are far from people’s residents it reduces peoples’ demand to it and therefore widen the inequity gap in the use of health care which has inversely health outcomes.

**Fig. 4:**
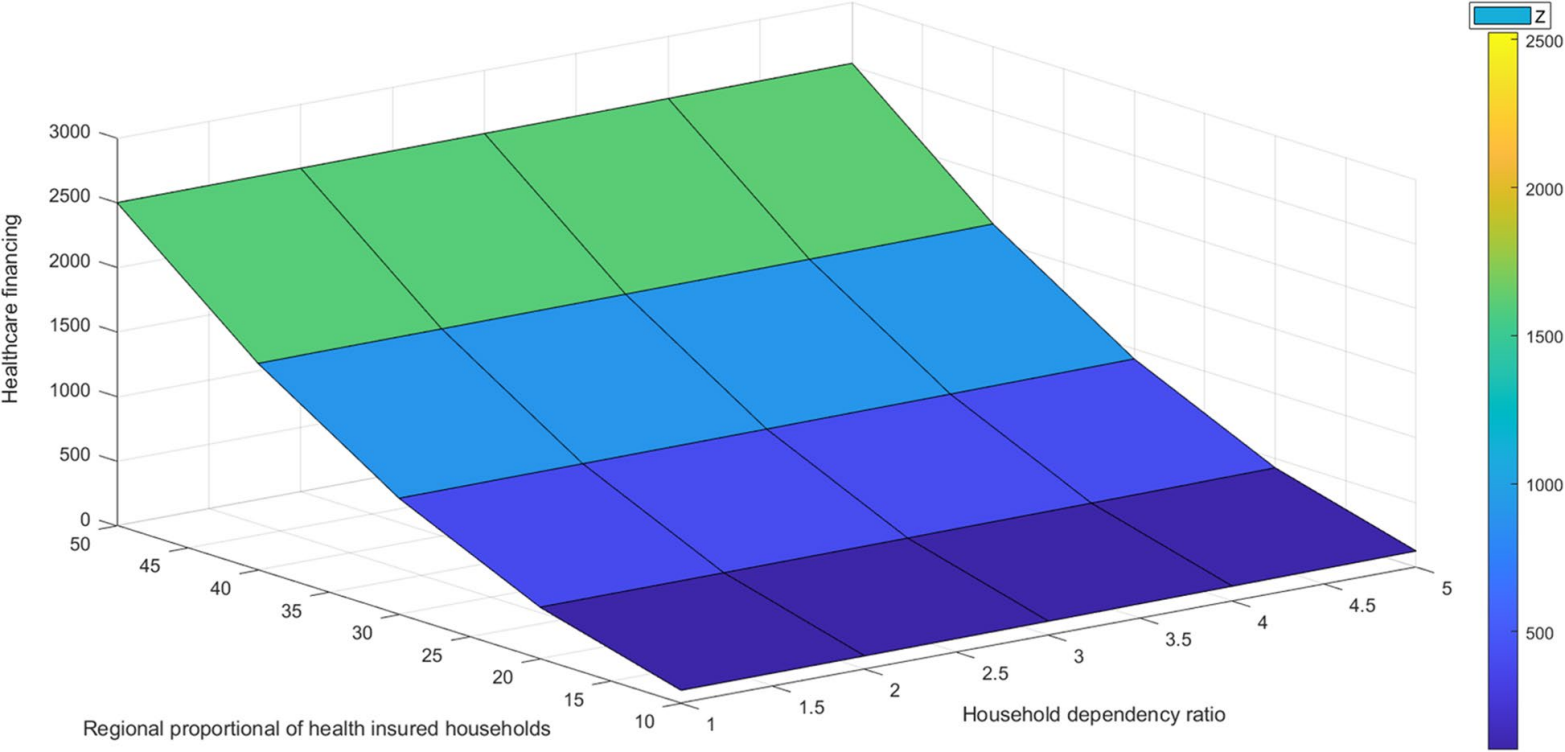
Healthcare financing, health insurance and household dependency ratio

Results on Fig. 4 show that, the number of household dependency ratio has negative effects to the household health insurance proportion, while it has positive relationship with the household healthcare financing which implies that when households have large number of dependents their health care financing is relatively high. The cost of health care becomes high as number of non-working members of household increases, the same affects the household chances to subscribe to social health protections [5, 32, 33].

### Effects of social health protection on household equity health care financing

Results in Table 6 show that having health insurance increases equity in household health care financing significantly across clusters (i.e., national level, rural and urban). This implies that health insurance is a good strategy in implementing in-country and global agenda towards the realization of the universal health care coverage. However, studies [4, 5] signal that when health insurance availability is characterized with perfect market structure it can destroy the public health insurance through major advertisement made by the private institutions. Moreover, leaving the public health insurance companies alone to dominate the sector can lead to the inefficiencies and frequently market failures as most government in the developing word have been taking funds/capital in these social funds and use it elsewhere in different project which has led to the failure and collapse of many social health protection funds [1, 6, 17].

**Table 6:** Full information maximum likelihood estimates of the endogenous switching regression model for household health care financing

**Variables**	**Model estimates**		
	**Selection equation** **(1)**	**Rural** **(2)**	**Urban** **(3)**
Sex	0.162*** (6.219)	0.070** (2.902)	0.103* (2.94)
Age	-0.210 (0.244)	0.190 (0.275)	0.1702 (0.242)
Household size	-0.009** (2.755)	-0.032** (3.278)	-0.059*** (5.031)
Marital status	0.126 (0.406)	0.035 (0.685)	0.056 (0.573)
Education	-0.468** (2.674)	-0.366*** (6.672)	-0.427** (4.383)
User fee	-0.127** (2.571)	-0.321** (3.043)	-0.451** (5.003)
Out of pocket	-0.035 (1.06)	-0.001 (0.216)	0.018** (2.015)
Household income	0.184*** (3.571)	0.053*** (3.063)	0.051*** (5.040)
Distance to health facility	0.175 (1.082)	0.146 (0.548)	0.316 (1.034)
Employment	0.059** (2.532)	0.192** (4.657)	0.317 (7.583)
Sickness	-0.129*** (4.268)	-0.481*** (7.842)	-0.106** (1.998)
Household dependence ratio	-0.152*** (4.878)	-0.303** (3.519)	-0.189*** (5.783)
Social health protection (Insurance)	0.223** (3.672)	0.305*** (5.892)	0.389031*** (7.153)
**Model diagnostics**			
Wald chi2(10)	174.16		
Prob > chi2	0.0000		
Log-likelihood	-6787.184		
Number of observations	34,567	23,562	11,005
LR test	Chi 2(1) = 0.005	Prob > chi2 = 0.6329	

*Absolute values of Z statistics in parenthesis*

^***^ p < 0.01, ** p < 0.05, * p < *0.1*

Moreover, results in Table 6 show that socioeconomic factors such as sex, household size, dependency ratio, education and employment status of the head of households influence equity in household health care financing significantly. Studies [2, 6, 9] concur with these findings and suggest that, the existence of rural to urban differences may increase inequities in the distribution of medical resources, of which rural residents are more likely to bear bigger burden due to diseconomies of scale associated with localities.

The endogenous switching regression model produces mean outcome on treated households under the study and their corresponding counterfactual outcomes which explains effects of the outcome if there were no any categorization of households into two groups. The average treatment effect on treated (ATT) is therefore a net difference between these two outcomes. Similar to that, ESR also produces mean outcomes for control groups of which in our study is the urban households and its counterfactual; and the difference between these two outcomes is referred as average treatment effect on untreated (ATU) which have been presented in Table 7.

**Table 7:** ATT and ATU of households in clusters (rural vs urban): ESR estimates

Outcome	Mean outcomes		Treatment Effects		Effects (%)
	Rural	Urban			
Health care financing	17.899 (0.874)	14.815 (0.731)	ATT	3.731*** (0.373)	14.7
	16.223 (0.867)	13.592 (0.619)	ATU	2.529*** (0.305)	24.8

*Standard errors in parentheses*

^***^ p < 0.01, ** p < 0.05, * p < *0.1*

Therefore, results in Table 7 show that the treatment effects estimation of households in rural areas on health care financing is positive and significantly different from zero with the value of the ATT being 3.731. These results implies that households being in rural areas significantly increasing households’ equity in health care financing by 14.7 percent while those of urban increases by 24.8 percent. The difference implies that those in urban areas are more likely to enjoy equity in health care financing compared to those in rural areas.

## Conclusion

The study has shown that most of the household’s health related costs such as user fees and out of pocket expenditure are major hindrance towards the realization of the equity in health care financing across households in rural and urban areas. Although these costs are considered as important part of the health care financing, they deplete significant share of households’ income which make them vulnerable to poor health outcomes and poverty, to which rural residents are highly affected compared to their urban counterparts.

Moreover, the increased investment on the social health protection across rural and urban residents in most of the developing countries are frequently hindered by the long-standing market and government failures through the nature of social health protection offered by the public and private sectors. For instance, when the public health insurances are cheap yet they cannot cover most of the health care treatments and medication making most of the urban residents reluctant to subscribe to these schemes, on the other hand due to health market competition most of the private insurances companies with great advertisement power destroys the reputation of the public insurances and reduces majorities demand to cheap public health insurances, which has adverse effects on the government efforts of increasing equity in health by deteriorating peoples’ ability to access and utilize health care.

Practically this study informs public and policy makers on the necessity of ensuring equitable distribution of health and non-health resources in order to improve welfare of the rural residents and hence improved health outcomes. This should hand in hand with increasing public expenditures on the construction of health facilities and installment of a highly needed medical instrument in order to bring all important health services closer to people’s residents.

### Limitation of the study

Although the study has managed to provide potential information for the improvement of the health sector in a country, it is not exempted from common methodological and data limitations especially when the study has utilized the secondary data particularly the panel data [34]. In most studies measuring equity has been a difficult task across economists therefore in this study the categorical measurement adopted may have some limitations because respondent’ decisions are sometimes based on their knowledge or psychological stance which have powerful effects on the information collected. Thus, for the secondary data it is difficult to control these issues because data have been collected by other researchers or authorities [35, 36].

On the other hand, most of the time-invariant variables such as sex which is not changing with time while other factors are changing, therefore this may implicate the study which may cause difficultness in accounting for statistical model. Moreover, just like any other secondary data, panel are normally subjected to problem of data quality especially those caused by the measurement errors, missing information and presence of outliers which affects the quality of results.

### Summary and area for further studies

In summary, the study has shown that an increase of the health care costs increases inequity in health care financing among households while health insurance is vital for relaxation of these effects as it significantly increases equity and lowers household health care burden. Moreover, equity in health care financing and social health protection were found to vary significantly across rural and urban residents indicating that residence has intermediate effects on the health equity which was found to be perpetuated by the economies and diseconomies of one staying in any of these two areas.

Moreover, the study recommends future studies to use the World Health Organization (WHO) indicators of equity in analyzing adoption and extent of social health protection among household in developing countries.

## Data Availability

Data will be available upon reasonable request.
